# Secondary breast reconstruction using a cross-chest profunda artery perforator flap in a vessel-depleted chest: A case report

**DOI:** 10.1016/j.jpra.2026.04.026

**Published:** 2026-05-04

**Authors:** Thierry Schweizer, Dariush Nikkhah, Shadi Ghali

**Affiliations:** a Department of Plastic Surgery, Royal Free Hospital NHS Foundation Trust, Pond Street, London, United Kingdom; b Department of Plastic, Reconstructive, Aesthetic and Hand Surgery, University Hospital Basel, Spitalstrasse 21, Basel, Switzerland

**Keywords:** Microsurgery, Breast reconstruction, Radiotherapy, Salvage procedure, PAP flap, Free flap

## Abstract

**Background:**

Secondary autologous breast reconstruction is technically demanding, particularly in patients with depleted or compromised recipient vessels following prior surgery and radiotherapy.

**Case:**

We report a case of delayed unilateral breast reconstruction using a profunda artery perforator (PAP) flap anastomosed to contralateral internal mammary vessels after failure of primary DIEP flap reconstruction and radiation-induced ipsilateral vessel occlusion.

**Results:**

A double-perforator PAP flap with a 12-cm pedicle enabled tension-free presternal tunneling and successful microsurgical anastomosis. Indocyanine green angiography confirmed adequate perfusion. Postoperative recovery was uneventful, and follow-up demonstrated a stable reconstruction with a satisfactory aesthetic outcome.

**Conclusion:**

Cross-chest PAP flap reconstruction is a feasible and effective strategy in vessel-depleted chests and should be considered when conventional reconstructive options are exhausted.

## Introduction

Secondary breast reconstruction presents unique technical challenges, particularly when recipient vessels have been compromised by previous surgery or neo-adjuvant radiotherapy. Breast reconstruction using free tissue transfer remains the gold standard for achieving durable and natural aesthetic outcomes, particularly in patients who have received radiotherapy.^1^^,^^2^ The deep inferior epigastric perforator (DIEP) flap is the most commonly used option; however, prior abdominal surgery, unfavorable anatomy, or pervious intraoperative failure may preclude its use.

In such cases, thigh-based flaps, particularly the Transverse Upper Gracilis (TUG) and Profunda Artery Perforator (PAP) flaps, represent reliable secondary options. Recent evidence demonstrates that PAP flaps offer comparable success rates with significantly fewer vascular complications and unplanned reoperations compared with TUG flaps, as well as a longer pedicle, which may be advantageous in complex reconstructions.^3^ Larger contemporary series have further confirmed the safety, reproducibility, and high patient satisfaction associated with PAP flap breast reconstruction.^4^

When ipsilateral recipient vessels are not available due to prior surgery or radiation, the use of contralateral internal mammary vessels via a presternal tunnel has been described as a salvage strategy in microsurgical breast reconstruction with DIEP flaps.^5^^,^^6^ However, reports combining this approach with PAP flaps remain scarce. We present a case illustrating the successful application of a cross-chest PAP flap in a vessel-depleted chest.

### Case report

A 43-year-old woman underwent a left skin-sparing mastectomy with axillary lymph node clearance and attempted immediate autologous reconstruction using a DIEP flap 3 years ago. Intraoperatively, the deep inferior epigastric artery (DIEA) was found to be of small caliber with significant mismatch to the recipient vessels. Multiple arterial and venous anastomotic attempts were performed, including four to the internal mammary vessels and two to the lateral thoracic vessels, all of which failed to establish adequate blood flow. The reconstruction was therefore abandoned, and the mastectomy site was closed.

Following adjuvant chemo- and radiotherapy, delayed reconstruction was planned. Preoperative computed tomography angiography demonstrated occlusion of the left internal mammary and thoracodorsal vessels, a recognized sequela of radiation-induced vascular injury.^1^^,^^2^ Bilateral lower-limb imaging confirmed suitable PAP and TUG perforators. Owing to the anticipated need for a longer pedicle to reach contralateral recipient vessels, a PAP flap was selected.

**Figure 1:**
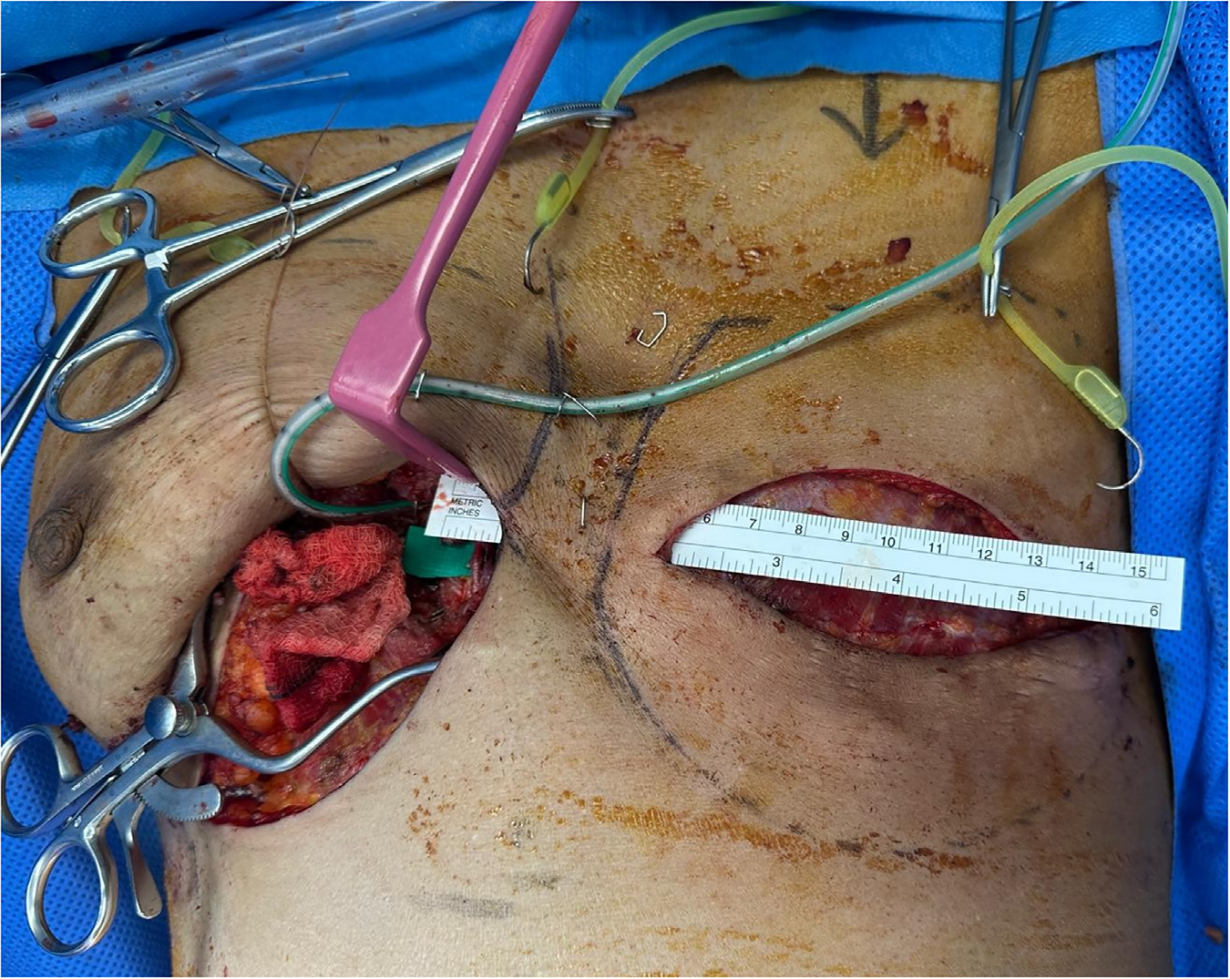
Intraoperative photo of contralateral submammary incision (right breast) und subcutaneous presternal tunnel for pedicle. Right internal mammary vessels are highlighted with a green background sheet.

A left double-perforator PAP flap was harvested, with a pedicle length of approximately 12 cm, consistent with previously reported anatomical advantages of the PAP flap.^3^ The flap was tunneled subcutaneously superficial to the sternum to the contralateral right chest without tension (Figure 1). Microsurgical anastomosis was performed to the right internal mammary vessels. The venous anastomosis required revision due to torsion. Despite the friable nature and small caliber of the recipient vessels, arterial inflow and venous outflow were successfully established. Intraoperative indocyanine green angiography confirmed complete flap perfusion.

**Figure 2:**
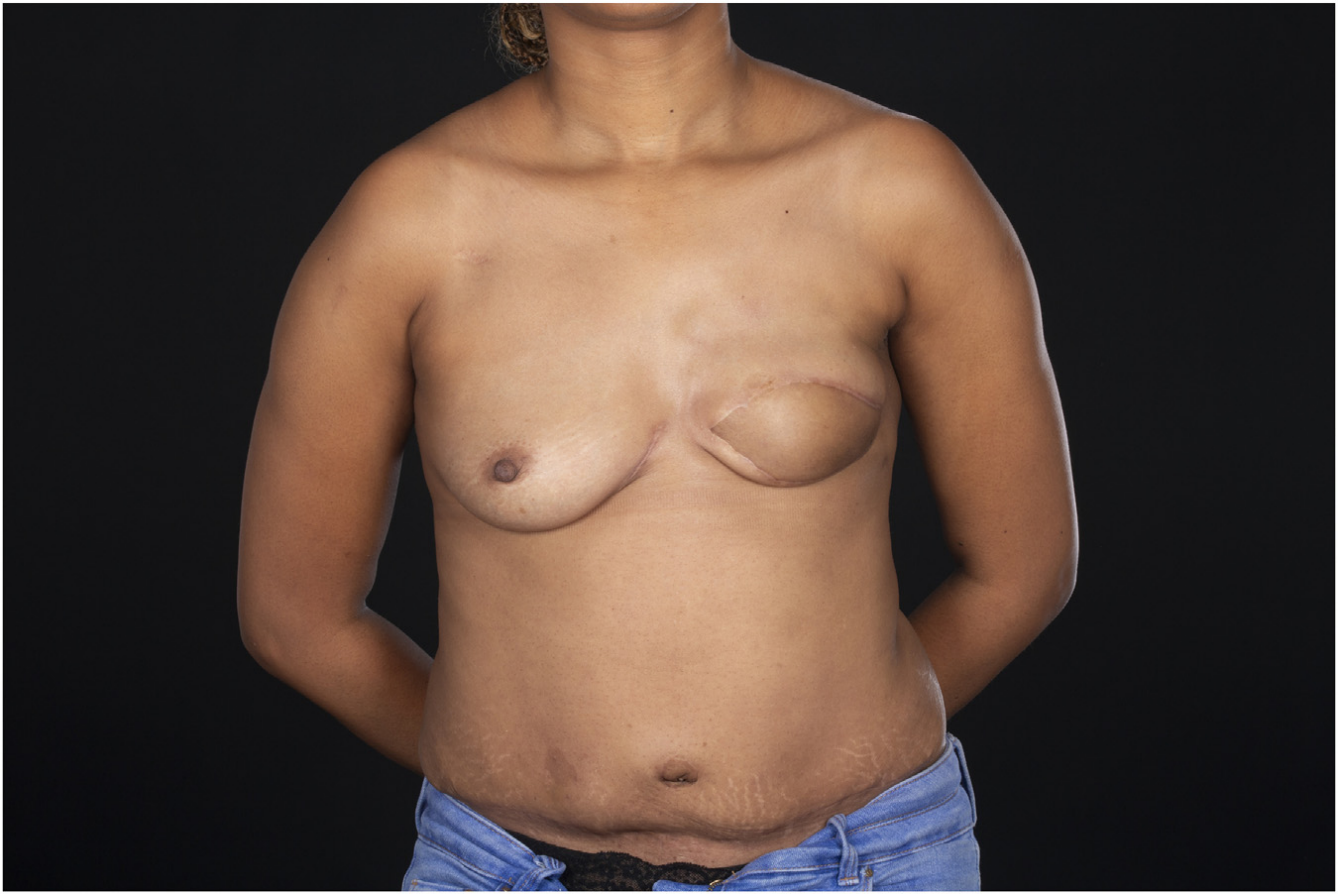
11 months postoperative result after left sided secondary reconstruction with a PAP-Flap to the right IMA vessels.

The postoperative course was uneventful, with no evidence of vascular compromise or wound complications. Clinical follow-up at 11 months demonstrated a stable reconstruction with an good aesthetic outcome (Figure 2). The patient is currently scheduled for nipple reconstruction.

## Discussion

Secondary autologous breast reconstruction following failed primary reconstruction and radiotherapy remains one of the most challenging scenarios in microsurgery. Radiation-induced endothelial damage, fibrosis, and vessel fragility significantly increase the risk of intraoperative complications and limit the availability of reliable recipient vessels.^1^^,^^2^ In this context, both flap selection and recipient vessels must be carefully assessed and chosen.

The PAP flap has emerged as a reliable secondary option when the abdomen is unavailable or unsuitable. Since its original description for breast reconstruction by Allen et al.,^7^ the PAP flap has demonstrated consistent anatomy, adequate vessel caliber, and a favorable donor-site profile. Larger contemporary series confirm high flap success rates, low donor-site morbidity, and excellent patient-reported outcomes.^4^ Compared with TUG flaps, PAP flaps are associated with significantly fewer vascular complications and fewer unplanned reoperations, while offering a longer pedicle length.^3^^,^^8^ These characteristics make the PAP flap particularly well suited for complex secondary reconstructions requiring alternative recipient vessels.

Recipient vessel depletion is a recognized problem in patients who have undergone prior surgery and radiotherapy. When ipsilateral internal mammary or thoracodorsal vessels are unavailable, several salvage strategies have been described, including the use of alternative recipient vessels, vein grafts, or contralateral internal mammary vessels.^9^ Cross-chest anastomosis to the contralateral internal mammary system via a presternal tunnel has been reported mainly in DIEP flap reconstruction, using either antegrade and retrograde flow or venous crossover bypasses.^5^^,^^6^ These techniques demonstrate that contralateral internal mammary vessels can provide reliable inflow in vessel-depleted chests, albeit with increased technical complexity.

In the present case, the long pedicle length of the PAP flap allowed direct cross-chest anastomosis without the need for interpositional vein grafts, reducing additional anastomoses and potential thrombosis risk. This advantage is particularly relevant in irradiated patients, where vessel fragility may compromise graft patency.

Preoperative computed tomography angiography was essential in this case, confirming both donor-site perforator suitability and ipsilateral recipient vessel occlusion. Such imaging is critical in secondary reconstruction to guide flap choice and operative planning, particularly when unconventional recipient vessels are anticipated.

To our knowledge, reports of cross-chest PAP flap breast reconstruction remain scarce. This case adds to the growing body of literature supporting flexible recipient vessel strategies and highlights the PAP flap as a valuable tool in the reconstructive armamentarium for vessel-depleted chests.

## Conclusion

Cross-chest profunda artery perforator flap reconstruction is a feasible and effective option for secondary breast reconstruction in vessel-depleted chests. The combination of a reliable donor site, long pedicle length, and alternative recipient vessel selection can facilitate successful outcomes in highly complex reconstructive scenarios.

## Funding

None.

## Patient consent

The patient provided informed consent for the surgical procedure and publication of this case.

## Reporting guidelines

This article is written in adherence with the STROBE Guidelines.

## Prior meeting

This work has not been presented at any meeting.

## Declaration of competing interest

None declared.

## References

[1] Fracol M.E., Basta M.N., Nelson J.A. (2016). Bilateral free flap breast reconstruction after unilateral radiation: comparing intraoperative vascular complications and postoperative outcomes in radiated versus nonradiated breasts. Ann Plast Surg.

[2] Prantl L., Moellhoff N., von Fritschen U. (2021). Effect of radiation therapy on microsurgical deep inferior epigastric perforator flap breast reconstructions: a matched cohort analysis of 4577 cases. Ann Plast Surg.

[3] Borrelli M.R., Spake C.S.L., Rao V. (2023). A systematic review and meta-analysis comparing the clinical outcomes of profunda artery perforator versus Gracilis thigh flap as a second choice for autologous breast reconstruction. Ann Plast Surg.

[4] Atzeni M., Salzillo R., Haywood R., Persichetti P., Figus A. (2022). Breast reconstruction using the profunda artery perforator (PAP) flap: technical refinements and evolution, outcomes, and patient satisfaction based on 116 consecutive flaps. J Plast Reconstr Aesthet Surg.

[5] Tyrell R., Leong R.Y., Sathyanarayana S.A., Korn P., Kadison A.S. (2016). Using a single internal mammary artery as retrograde and antegrade flow for bilateral deep inferior epigastric artery perforator reconstruction: a case report. Int J Angiol.

[6] Cai A., Geierlehner A., Arkudas A., Horch R.E. (2022). Bilateral free flap breast reconstruction using venous cross-over bypass to contralateral internal mammary artery for salvaging thrombosed arterial anastomosis in unilateral repeated irradiation of the breast. Microsurgery.

[7] Allen R.J., Haddock N.T., Ahn C.Y., Sadeghi A. (2012). Breast reconstruction with the profunda artery perforator flap. Plast Reconstr Surg.

[8] Hunter J.E., Lardi A.M., Dower D.R., Farhadi J. (2015). Evolution from the TUG to PAP flap for breast reconstruction: comparison and refinements of technique. J Plast Reconstr Aesthet Surg.

[9] Cohen-Hayoun E., Pessis R., Lkah C., Atlan M. (2013). Strategies in case of unusable internal mammary vessels in a mammary reconstruction by DIEP. Ann Chir Plast Esthet.

